# “I Like the One With Minions”: The Influence of Marketing on Packages of Ultra-Processed Snacks on Children's Food Choices

**DOI:** 10.3389/fnut.2022.920225

**Published:** 2022-07-22

**Authors:** Priscila de Morais Sato, Fernanda Helena Marrocos Leite, Neha Khandpur, Ana Paula Bortoletto Martins, Laís Amaral Mais

**Affiliations:** ^1^Department of Nutrition Sciences, Federal University of Bahia (UFBA), Salvador, Brazil; ^2^Department of Nutrition, University of São Paulo (USP), São Paulo, Brazil; ^3^Brazilian Institute for Consumer Defense (Idec), São Paulo, Brazil

**Keywords:** ultra-processed foods, snacks, children, food label, food marketing, focus groups

## Abstract

**Objective:**

This study aimed to assess the most consumed school snacks using the free listing and understand how marketing strategies on food labels influenced children's perceptions of snacks *via* focus groups.

**Design:**

The study design involved free lists and semi-structured focus group interviews.

**Setting:**

São Paulo, Brazil.

**Participants:**

A total of 69 children were involved in this study.

**Phenomenon of Interest:**

Children's perceptions of food labels.

**Analysis:**

Food groups mentioned on the free lists were analyzed for their frequency and priority of occurrence. The focus groups were analyzed through content analysis.

**Results:**

Juices and chips were the most salient snacks, with availability and flavor as reasons for their consumption. Children found images on labels appealing, which created a desire for the food, although could be deceptive. Snacks perceived as healthy were encouraged by parents, and children could more easily convince them to buy snacks with health claims. Colors and brands were important to catch children's attention and make the snack recognizable. Television commercials and mascots reinforced marketing strategies on labels.

**Conclusions and Implications:**

Our results point to the need for public health strategies to deal with the obesity epidemic through creating and implementing specific legislation to regulate food labels to discourage the consumption of unhealthy snacks and prohibit food marketing targeted at children.

## Introduction

Food marketing refers to any form of commercial communication or message that is designed to increase the recognition, appeal, and consumption of particular food products (1). It involves a set of persuasive and sophisticated techniques (2) used by food manufacturers to promote their products through different vehicles of promotion, like television, social media, and product packing, *via* varying marketing strategies, including product placement and design (3). In this context, food packages serve as a powerful marketing tool at the point of purchase (4). Visual and informational front-of-package marketing cues constitute salient elements of the food environment that may influence consumers' decisions on what to buy, what to eat, and how much to eat (5, 6).

Several marketing strategies on food packages are targeted specifically at children and adolescents (7). This is because first, frequent marketing exposure to food promotions at earlier stages of life can contribute to a strong positive effect toward specific brands and products and create loyal consumers in the future (8), and second, food advertising causes “pestering” by children. “Pester power” is defined as the children's influence over adult purchasing through demands and requests and has been associated with parents buying less healthy foods (9, 10).

The most common techniques used on food packages to persuade children include compelling graphic elements like the use of bright colors; promotional, licensed, or cartoon characters; celebrity endorsers; sportspersons; graphic references to fun and play; and premium collectible offers with toys and child-friendly lettering (11). A systematic review of the persuasiveness of front-of-package marketing cues on food packages for children has shown that this audience was more likely to choose a product that has an endorser and/or illustrations (6). Similarly, Elliot (12) found that children from 6 to 12 years of age were strongly influenced by package designs featuring characters, particular colors, and pictures of the product (12).

Child-oriented marketing strategies are commonly used on labels of ultra-processed food products (UPFs) (13–15) that are positively associated with obesity (16, 17) and a range of other non-communicable chronic diseases (NCDs) (18–21). UPFs are ready-to-eat or heat formulations made by assembling food substances (e.g., sugars, oils and fats, proteins, starches, and fibers) and “cosmetic” additives (e.g., flavors, flavor enhancers, colors, emulsifiers, and sweeteners) through a series of industrial processing. They are highly profitable branded products distributed on an industrial scale with a poor nutritional profile (22, 23). Given their convenience and palatability, these products are frequently marketed to and consumed by children as snacks at school (24).

In Brazil, data from the National Survey of Schooler Health (*Pesquisa Nacional de Saúde do Escolar* – PeNSE) conducted in 2019 with 11,851,941 schoolers showed that 97.3% of schoolers had consumed UPFs the day before, with crackers being the most consumed snack (49.3%), followed by cookies (46.8%), breads (42.0%), and sodas (40.8). Although 75.3% of participants affirmed that the school offered meals, 48.0% of participants never or rarely ate them. Additionally, the consumption of foods from school canteens and informal selling points in the schools' surroundings were reported by 48.8 and 48.7%, respectively. Foods and drinks most available in canteens were baked goods, fruit juices, and sodas. In informal selling points, they were sodas, crisps, and deep-fried goods (25).

There is growing evidence demonstrating that the marketing of energy-dense and nutrient-poor foods can negatively influence children's food attitudes, preferences, and consumption, leading to adverse health outcomes (26). A recent systematic review carried out by Smith and colleagues (3) reinforced the detrimental effects of food marketing techniques aimed at children and adolescents from 0 to 18 years, particularly those used in TV/movies and product packaging. However, the review also highlighted a lack of qualitative research that investigates children's opinions about food labels and how they interpret information presented; only 3 out of 71 studies identified applied qualitative methods (3). Moreover, only two studies were not conducted in the global north, although childhood overweight and obesity are major concerns in most global south countries. To fill this gap, the objective of this study was to assess how children in São Paulo, Brazil, perceived labels of ultra-processed snacks that they found most appealing. Specifically, this study aimed to (1) highlight the food and drink items mostly consumed as snacks at school using free lists and uncover the rationale behind the choices and (2) understand how marketing strategies on UPF labels influenced children's perceptions of snacks.

## Methods

### Study Design

We conducted a qualitative study with focus groups (FGs) that incorporated a free lists exercise. FG is a research technique that produces data through interaction. The free lists exercise aimed to complement information produced from FG discussions by eliciting the most relevant food and drink snacks for children in the school context. Materials and methods are reported below, following the Standards for Reporting Qualitative Research (SRQR) (27) and the Consolidated Criteria for Reporting Qualitative Research (COREQ) (28).

### Sampling and Setting

A total of 69 participants provided data that were used in this study. Participants were recruited by convenience, through a Brazilian research firm, from a database of potential participants. Participants were approached by email, and the inclusion criteria were as follows: (1) ages between 7 and 12 years, (2) living in urban regions of São Paulo municipality, and (3) agreeing to participate and having parental consent. The exclusion criterion was having parents working in the health sector and/or in the food or tobacco industries. Nobody refused to participate in the study, and no participants dropped out. None of the authors had any interaction with the participants prior to or during the FGs. Participants had no information about the researchers or the FG moderator. Nine FGs were conducted during the day, between 1 and 8 August 2019, in a research facility in São Paulo, Brazil.

The FGs were stratified by age range (7–9 and 10–12 years) and socioeconomic status (SES) (A+B1 and B2+C). Stratification was justified as follows: (1) children in the same FG are recommended not to be more than 2 years apart in age (29) and (2) SES could affect food availability and the experiences and perceptions with food labels (30, 31). SES was assessed prior to the children being recruited according to the criteria based on the households' possession of goods proposed by the “Brazilian Association of Research Companies” (30). The criteria covered the following: (1) the number of bathrooms, domestic employers, automobiles, personal computers, dishwashers, refrigerators, freezers, washing machines, DVD players, microwave ovens, motorcycles, and clothes driers in the household; (2) the householder education; and (3) the access to public utility services (piped water and paved street). Based on the responses, the household is classified from A (high SES) to E (low SES).

### Ethics Committee

Procedures involving research study participants were approved by the Research Ethics Committee of the Public Health School at the São Paulo University (protocol no. 3.441.247). Written informed consent was obtained from all participants' caretakers, and verbal assent was obtained from all participants before the focus groups.

### Data Collection

We used data triangulation of FG and free lists to assess food and drink items mostly consumed at school by the participants. The listing activity and the FG guide were pretested with a group of children aged 7–9 years, B2+C SES. These data were not included in the final sample. In our study, foods and drinks considered “desired snacks to eat at school” were expected to be relatively similar across participants, as: (1) children's eating practices are highly influenced by their peers (32), (2) classification of foods as acceptable to compose a specific meal (as a snack to eat at school) is culturally shared (33, 34), and (3) desirability of foods are products of social interactions (35). Most foods and drinks considered appropriate snacks to eat at school were likely to be common across participants, allowing for the identification of the most salient products.

All free lists and FGs were conducted in Portuguese, by a female, trained moderator who graduated in social sciences and who specialized in data collection with children. At the time of the study, the moderator was employed by the Brazilian research firm responsible for recruiting the participants, having vast experience in conducting FGs. Also participating in FGs were one note-taker and one to three observers behind a one-way mirror who were also taking notes.

For the free lists exercise, at the beginning of FGs, after an ice-breaker question (“What shows or cartoons do you like to watch?”), we asked participants to individually write a list of desired snacks to eat at school. Children were given 5 min to make a list as complete as possible. During the time taken to complete the task, the moderator ensured that the children were not communicating with each other or seeing each other's lists. The literature on free lists suggests that about 30 participants are needed to provide a representative sample (36), and this study had 69 participants.

After the lists were completed and collected (approximately 10 min), the moderator started the FG discussion. Based on the foods and drinks presented in the free lists, the interview guide approached: (1) snacks (foods and drinks) consumed at school, (2) reasons for and frequency of consuming them, and (3) opinions and perceptions about the food and beverage packages. The average FG duration was 1 h and 5 min. The audio was recorded and transcribed verbatim. Transcripts were read by PdMS, and data saturation was considered reached after eight FGs, meaning that no relevant new information was identified in the last FG (37).

### Analysis

Food lists were analyzed for the most salient foods and drinks using the data analyses software Free-List Analysis under Microsoft Excel (FLAME v1.1). First, the foods and drinks listed were grouped into 12 broad food categories by two researchers (PdMS and NK). In the analysis, triangulation of free lists and FG was used to inform free list categories based on children's own food classification during FG discussions. As not all children described foods' characteristics with the same specificity, grouping foods served to unify similar foods. For instance, one child wrote “natural juice” and the other “juice,” hindering UPF classification. In cases in which the food or drink's process level was not clear, triangulation of data allowed consulting FG discussions to assess more detailed information about the item. Also, categories did not distinguish between similar snacks with different food flavors and brands (e.g., grapes and apples were classified as fruits).

Food groups were analyzed for their frequency of occurrence, in addition to Smith's S salient measure. This measure was computed based on the number of times each food and drink was mentioned and how much priority they were given in the lists (i.e., mentioned first vs. lower down): *S* = [(*L* – *Rj* + 1)/*L*]/*N*, where *L* is the length of each list, *Rj* is the rank of item *J* in the list, and *N* is the number of lists in the sample (38). Thus, the salience analysis did not allow high prioritization of an item rarely mentioned by most participants, allowing a better representation of the whole group's perspectives on an item's importance (39).

The FG discussions were analyzed using content analysis, as described by Bernard, Wütlich, and Ryan (40). We used an inductive approach, which allowed new codes to emerge from our data. In this step, data triangulation contributed to the creation of the codes related to the most liked snacks to eat at school, as results from the free lists confirmed the relevance of the emergent codes. The triangulation of researchers during the coding process aimed to aggregate multiple views and increase the robustness of the analysis. First, one researcher (PdMS) read all transcripts, highlighting important information and making memos. Exploratory coding was performed using a cutting and sorting approach, in which similar information was grouped together, forming sets of emergent codes. A codebook was built that included, for each code, short and detailed description, inclusion and exclusion criteria, typical and atypical examples, and an example named “close but no,” which illustrated the code's limits. The codebook was applied by two other researchers (LAM and FHML) to all transcripts. Agreement between coders was assessed through Cohen's kappa coefficient (41) for inter-rater reliability (kappa = 0.91). All analyses were performed using MAXQDA 2020.

## Results

A total of 69 children composed the nine FGs. More details about the participants' age range, SES, gender, and type of school are presented in Table 1.

**Table 1:** Characteristics of the participants (*n* = 69).

**Children's characteristics**	**Number of participants, *n* (%)**
Age range (years)	
7–9	30 (43.5%)
10–12	39 (56.5%)
SES	
A+B1	31 (44.9%)
B2+C	38 (55.1%)
Gender	
Male	35 (50.7%)
Female	34 (49.3%)
Type of school	
Public	27 (39.1%)
Private	42 (60.9%)

*São Paulo, 2019*.

*SES, socioeconomic status; A+B1, high SES, according to the Brazilian Criteria; B2+C, low SES, according to the Brazilian Criteria*.

Food groups identified through free lists were juices, chips, fruits and vegetables, cakes, sodas, bread, milk and yogurt, cookies and crackers, candies and chocolates, fast foods, and water and coconut water. The 30 codes produced through the content analysis were classified into 10 themes, five concerning ultra-processed snacks consumed at school—highlighting what was most consumed, why (reasons for eating them) and how/when (based on rules associated with them); four about their packages and labels—concerning their design, marketing strategies, and participants' perceptions about them; and one about other media that reinforce labels' information—more specifically, about television commercials. The coding tree is presented in Table 2.

**Table 2:** Themes, subthemes, and codes that emerged through content analysis of nine focus groups in São Paulo, 2019.

**Themes**	**Subthemes**	**Codes**
Ultra-processed food and drink products consumed at school	Most liked snacks to eat at school	Juice
		Chips
		Cake
		Soda
		Breads
		Yogurt
		Cookies and crackers
	Reasons to eat it at school	Flavor
		Giving energy/satiating
		Healthy
		Others
	Food rules	Food rules
Packages and labels	Product brands	Product brands
	Design elements on the label	Colors
		Words
		Shapes
	Marketing elements on the label	Characters
		Information about the product
		Giveaways, games, and promotions
	Perceptions about the label	Feelings toward the food
		Labels' qualities
		Placement
		Changes
Other media that reinforce labels' information	Television commercials	Television commercials

### Ultra-Processed Food and Drink Products Consumed at School

Subthemes about UPF included *most liked snacks to eat at school* (juice, chips, cake, soda, breads, yogurt, and cookies and crackers) and *reasons to eat it at school* (flavor, giving energy/satiating, healthy, and others). How often children took a certain food or beverage to school was explained by *food rules*. Further details are described below.

**Figure 1:**
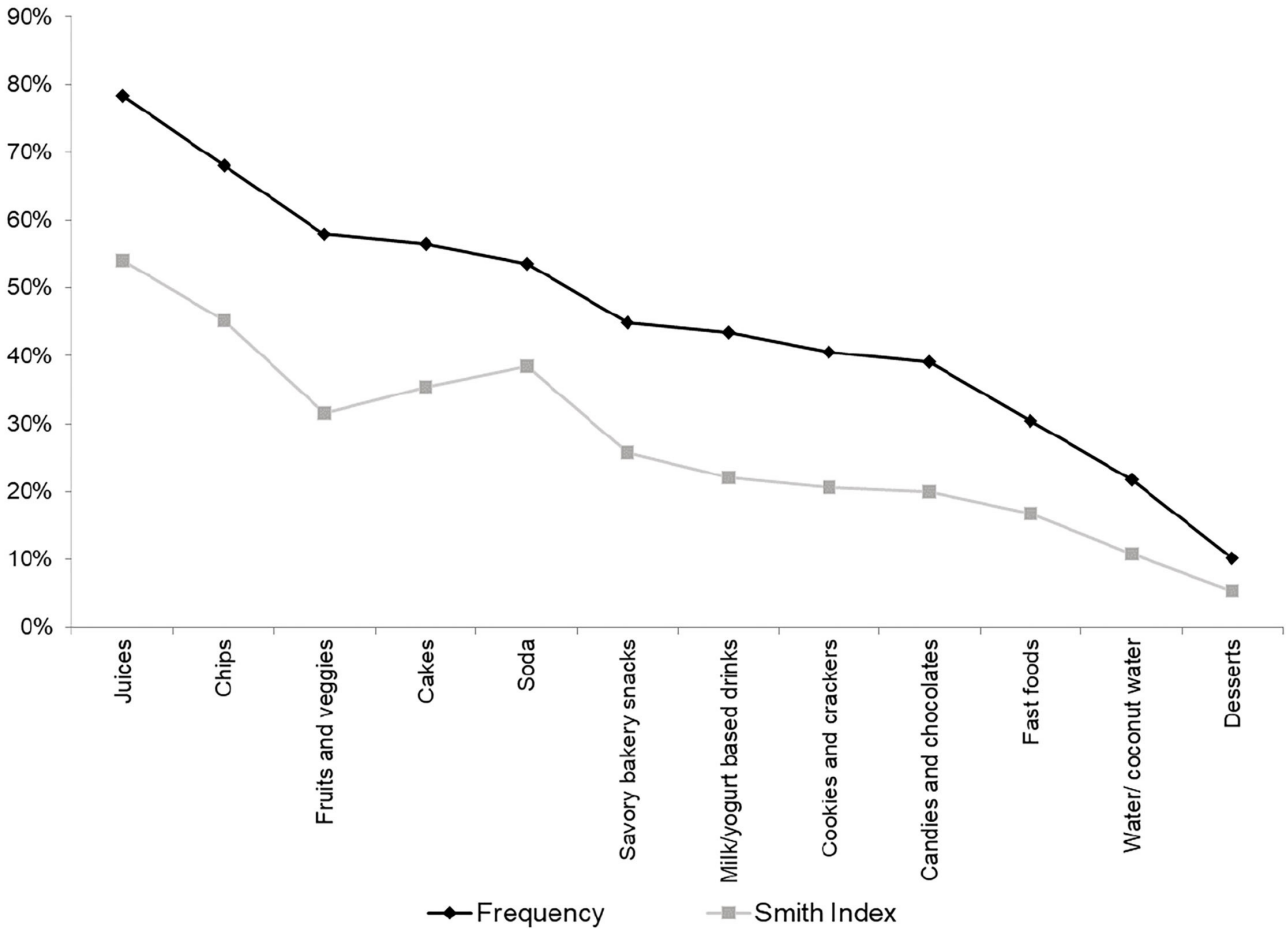
Free lists results by the frequency of the foods/drinks, São Paulo, 2019.

The main foods and beverages mentioned in the FGs reflected the most salient foods in the free lists exercise (Figure 1), which also included one non-UPF item—fruits and vegetables. However, Smith's index shows that, although they were frequently mentioned, they were included in the lists in lower positions, therefore not being the first choices thought by children.

Juice was the most mentioned food/drink in free lists and had the most coded segments. It was sometimes referred to as “packaged juice.” The main reason for drinking it at school was the flavor, followed by being considered healthy and giving energy. Other reasons were related to being refreshing. Its relevance in the context of school meals is reinforced by being the only food/drink most cited as “always eaten/drunk.” It was the second most accessible food/drink by some children in school, following sodas. However, unlike soda, juice was often served at school meals—“At my school, they give us juice” (7–9 years, A-B1).

Chips were the second most mentioned food/drink, mainly liked by children because of their flavor. Other reasons for taking it to school were being crunchy and giving energy/satiating. They were consumed at school sometimes, either brought from home or bought at the canteen. All other foods/drinks were also mainly consumed at school because of their flavors, except breads, which were consumed for giving energy/satiating. Cake and soda were the only foods/drinks to which being healthy was not a reason for eating/drinking them at school.

All food rules mentioned by children were related to eating healthy foods. Almost all rules were taught and set by their mothers, often being related to having specific days to eat what was considered “unhealthy”—“Friday is ‘cheat day', we call it that” (10–12 years, A-B1). Other contributors to perpetuating food rules were doctors—“I went to the pediatrician and she told me that I can't eat it [cookies and crackers] all the time otherwise I will have high blood pressure, this type of stuff, so I only eat it on weekends or Fridays” (10–12 years, B2-C)—and schools—“In my school we can't bring chips from Monday to Wednesday” (10–12 years, A-B1).

The foods/drinks most often linked with food rules were juice and soda. While sodas were unhealthy for having “a lot of sugar,” juices were “free” for children to drink—“My mom lets me take juice to school every day” (7–9 years, A-B1). In this case, the social rule was reinforced by peers where—“Almost everyone takes juice to school” (7–9 years, A-B1).

### Ultra-Processed Food Packages and Labels

Subthemes about UPF packages and labels included *product brands, design elements on the label* (colors, words, and shapes), *marketing elements on the label* (characters, information about the product, giveaways, games, and promotions), and *perceptions about the label* (feelings toward the food, labels' qualities, placement, and changes).

Product brands were frequently cited, especially cookies and crackers brands (24.29%), followed by sodas (17.14%) and chips (15.71%). Brands commonly replaced the food's name—“[I take to school] a bag of chips, a *Coke*® [soda brand]… but not every day” (10–12 years, B2-C)—or were used to indicate preference—“I really like *Natural One*® [juice brand], it is made from the fruit. It says so in the bottle” (10–12 years, A-B1). Alongside the brand names, children were well informed about other aspects of the product—“Mini *Oreo*® [cookie brand] has a new package and new flavors” (10–12 years, B2-C), being mentioned when images on the labels were described. The most cited image among brands were characters—“*Toddy*® [chocolate milk brand] has a little cow” (10–12 years, A-B1), followed by colors—“The [label] color that everyone knows is *Coke*® [soda brand]” (10–12 years, B2-C).

Overall, the most cited design element on the labels was related to color, followed by characters. Colors were mainly mentioned for being eye-catching—“The red is very flashy, strong color…” (10–12 years, A-B1), but children also recognized that they reinforced information about the product—“Sometimes the color distinguishes the flavor” (10–12 years, B2-C). Characters were mainly products' mascots—“A farmer with a yellow round face. He's the corn” (7–9 years, A-B1). They also included famous cartoons—“I like the ones with the *Minions*®” and personalities—“There is a soccer player saying that it's good. The package changes, but the player remains” (10–12 years, B2-C).

Marketing elements on the label depicted products' characteristics like foods' flavors, but also included ingredients—“[the chips] are made with actual potatoes” (7–9 years, A-B1). Information about ingredients also made the product very appealing—“The cookies packaging shows the chocolate drops… makes you drool” (10–12 years, B2-C). However, information was not always accurate—“Cookies… there is one with a nice photo on the package, but when you open it, it's like half a dozen of chocolate drops” (10–12 years, A-B1). Another main cited marketing element were surprise and collectible giveaways—“[this bag of chips] comes with a surprise, a sticker. You can collect the stickers, my cousin does” (7–9 years, B2-C), games—“Sometimes I see it [puzzle] and I solve it before drinking the juice. I solve the puzzles in the ones [food packages] I know that have it, as *Yakult*® [fermented milk drink brand] with the *Sponge Bob*®” (10-12, A-B1), and promotions—“*Ruffles*® [chips brand] has a promotion that they take you on a trip” (10–12 years, B2-C). Packages and label formats, and the words on them, were much less mentioned. The expiration date and ingredients were also mentioned by the children.

Most perceptions around packages and labels concerned feelings toward the food, which included it being flavorful and the desire to buy or eat it—“There is a picture that makes your mouth water… makes you want to eat it” (7–9 years, A-B1). One child mentioned craving the food very badly—“I got anxious because I saw the package and it had many *Doritos*® [chips brand], all those colors… I wanted to eat it right away” (7–9 years, A-B1). The second most mentioned perception was related to the label's qualities, in which children classified food labels, as eye-catching and appetizing, but, for some, “usually deceptive” (10–12 years, A-B1). Finally, during the conversation about the information on food labels, children mentioned another media that reinforced the information presented on food labels, “television commercials”—“There are TV commercials with the mascot” (10–12 years, B2-C).

## Discussion

Our study focused on children's most salient and desirable snacks to be eaten at school, unveiling the centrality of UPF, with the most frequently reported being juices and chips. The main reason for choosing a food/drink for a snack was flavor (as exemplified by the chips, the most liked salty snack), followed by giving energy/satiating and being healthy (as exemplified by juices, which were seen as healthy). Health concerns were taught by the adults, with children perceiving that they could rely on health claims to convince parents to allow them to buy/eat ultra-processed snacks. Different food characteristics were perceived by children to be promoted on food labels, influencing them to desire the product. Persuasive information about the products was transmitted by images, or even just by the product's brand, and was reinforced by other media, such as TV commercials.

While Letona et al. (42) have also described salty package snacks as one of the most reported purchased products among Guatemalan children, fruit drinks just appeared in fifth place, after sodas, candies, and pastries. In this sense, the presence of fruits and vegetables in third place in this study suggests a higher presence of *in natura*/minimally processed foods among our participants' school snacks. This observation highlights the importance of schools' food environment to children's food choices, as has been described by a systematic review conducted by Driessen et al. (43), and presents another mechanism through which schools may affect food choices and food rules. Thus, the amount of ultra-processed snacks consumed by children will be impacted by what is available to buy, what is given for free, and what is allowed to (bring to) eat there. This is particularly important considering the predominance of unhealthy snacks available in school canteens and food stores close to schools in Brazil (44, 45).

Corroborating Letona et al.'s (42) observations, our participants preferred snacks primarily because of their taste. Snack labels explore the foods' hyper-palatability, which is achieved in UPF through a myriad of additives (22). Thus, hedonic attributes competed with healthy ones to compose children's snack choices. The importance given to foods' healthiness in our study can be understood by the high presence of food rules in our participants' discourses, reinforcing the influence of nutrition education through parents/health professionals.

Claims related to health and nutrition have been described in food packages to attract children's attention in Guatemala (11), Uruguay (13), Canada (46), Australia (47), and Costa Rica (15). These studies show that, despite the claims, foods were classified as having low nutritional quality. When analyzing temporal tendencies, Elliott (48) described that, in Canada, child-targeted foods did not improve nutritionally over time, despite a significant increase of nutrition claims on their packages. However, health claims persuade consumers to incorrectly think that a food is healthier or that a product contains certain healthy foods (41). Our observations add to the current discussion on the persuasion effects of nutrition and health claims, as they did not only mislead parents' perceptions of foods but they also taught children erroneously which foods were healthy. Additionally, such claims were used by children to persuade their parents to buy the foods that contained them. Combined with the above-presented information, our results support the need of regulations that promote accurate information of the food's healthiness so food choices can be made more consciously by children and their caregivers.

In this study, children recognized strategies used by the food industry to make their products more appealing. In Uruguay, Gimenez et al. (13) have identified that bright colors and cartoon characters were the main marketing strategies among foods targeted at children. This resonates with the elements mentioned as important to our participants, suggesting the efficiency of these food marketing strategies. Similarly, Gamboa-Gamboa et al. (15) found that more than 40% of savory UPF snacks (n= 2,042) mostly consumed by Costa Rican children at school had at least one promotional character, with cartoons and company-owned characters appearing in 74% of them. Resonating with these observations, our participants mentioned games/puzzles as the main giveaways on food products. Although authors have described a diversity of promotions, including prizes (49), toys, and collectibles (11), games/puzzles might be convenient because of their low cost and easy access. However, this practice is worrying as it may induce children to build unhealthy food choices, as most foods with giveaways are ultra-processed (13) and the consumption of such foods starts being associated with fun. We suggest that the persuasive elements in UPF labeling highlighted in this study should be avoided even for the promotion of healthy snack options, as the reinforcement of attributes to increase the desire to eat is considered a way to take advantage of the lack of judgment of children under 12 years of age according to the Brazilian Consumers Defense Code (*Código de Defesa do Consumidor -* CDC) (50).

Our results add to the current understanding of children's food package perception by revealing the importance given to food brands by them and illustrating a high presence of brands from transnational companies in their discourses. We argue that current food packages not only promote products' hedonic and healthy attributes but also create and perpetuate an image related to a brand, including through brand-specific characters (11) that progressively become familiar and trustworthy (51). According to Aerts and Smits' (52) observations in Belgium, the unhealthier the food product targeted at children, the more marketing strategies there were on its package. Thus, marketing strategies on packages are also a vehicle to promote unhealthy and unsustainable foods and beverages that pose a global risk to people and the planet (53).

Additional strategies that reinforce the ones on food packages, such as TV commercials, were cited by our participants and resonate with Mehta et al.'s (47) study that described a cross-promotion of 77% of foods marketed to children in Australia. We highlight that the concomitant utilization of diverse marketing strategies on different media may reinforce the food's marketing message that, presenting the same identity throughout all media, is easy for children to recognize and identify themselves.

### Implications for Policy, Research, and Practices

In Brazil, the CDC already prohibits any kind of abusive marketing that takes advantage of the child's lack of judgment and experience (51). The Resolution n^o^163/2014 of the Brazilian Council for Children and Adolescents Rights (*Conselho Nacional dos Direitos da Criança e do Adolescente*- CONANDA) provides an interpretation of the CDC and the examples of abusive marketing, including the use of child characters, cartoons, promotions with awards or collectibles, excess of colors, etc (52). Despite the existence of legislation, the use of persuasive marketing strategies targeted at children is still quite common in Brazil, as shown by our results, demonstrating a lack of policy enforcement. In this sense, we highlight the need for specific regulation aimed to restrict marketing strategies of unhealthy food products, particularly those targeted at children, as well as sensitizing legal actors to this issue and raising consumers' awareness of their rights.

Latin America has examples of effective public policies in the last decade, combining the implementation of warning labels (black octagon) in foods with excessive amounts of critical nutrients together with the implementation of these warnings in all kinds of advertisements in Peru (53), the prohibition of advertisement directed to children in food labels with warnings in Chile (54) and México (55), and the ban on the sale of these products in and around schools in Chile (54). In Chile, where the implementation of a national law mandating front-of-package warning labels, restricting marketing, and banning school sales for products high in calories, sodium, sugar, or saturated fat began in 2016, scientific evidence has confirmed the reduction of purchases of high-in food products (50) and statistically significant reduction of sugar, sodium, and energy content of foods, especially dairy, confitures, and sugary beverages (54, 55). Considering these experiences, we point to the need of further research exploring the impacts of the package and label regulations in combination with other policies to restrict access to UPF in and around schools.

Health professionals and educators have a crucial role in promoting critical thinking about food marketing strategies. Actions targeted to children should focus on increasing children's advertising knowledge and help them engage critically with commercial messages in ways that are developmentally appropriate. Parents should be educated about food marketing along with the negative effects of high exposure to food marketing on children's food choices. Research on the area demands more engagement directly with young people to learn about the development of critical thinking across childhood (56).

Our study has some limitations. First, our results cannot be generalized to Brazil; however, we highlighted the heterogeneity of our sample in terms of SES and age, aimed to capture a diversity of children's views. Second, although not all food/drink snacks mentioned by children during FGs and the free lists were classified as UPF (22), we were able to focus on such foods by specifically exploring children's perceptions about product packaging and brands. Finally, as free lists were created simultaneously by all FG participants, one could worry about participants influencing one another doing it. However, the moderator was monitoring children at all times and assured no communication between the participants during the activity.

## Conclusion

Our study shows that Brazilian children preferred ultra-processed snacks at school, choosing them mainly because of their taste. Other valued foods' attributes were their ability to provide energy and healthiness, with the last being learned from parents/health professionals and explored as a marketing strategy on UPF packages. Marketing strategies used in foods and beverages targeted at children were mentioned by our participants, pointing to the efficiency of such elements in catching their attention and promoting snacks' hedonic and nutritional characteristics. In this scenario, there is an urge for public health measures to deal with the obesity epidemic by creating and implementing specific legislation to regulate packages and labels to discourage the consumption of unhealthy snacks, as well as to prohibit food marketing targeted at children, considering their lack of discernment and experience to understand commercial messages and to regulate the availability of unhealthy snacks in the school food environment, that is supposed to be safe for children.

## Data Availability Statement

The raw data supporting the conclusions of this article will be made available by the authors, without undue reservation.

## Ethics Statement

The studies involving human participants were reviewed and approved by Ethics Committee of the Public Health School from the São Paulo University. Written informed consent to participate in this study was provided by the participants' legal guardian/next of kin.

## Author Contributions

PS, FL, and LM performed the content analysis. NK performed the free list analysis. PS wrote the first draft of the manuscript. FL, AM, LM, and NK wrote sections of the manuscript. All authors contributed to conception, design of the study, revision, read, and approved the submitted version.

## Funding

Funding for this study was provided by UNICEF Brazil (BRZ/PCA/2017-2021/IDEC) and the São Paulo Research Foundation – FAPESP (Grants 2017/05651-0 and 2019/22278-7).

## Conflict of Interest

The authors declare that the research was conducted in the absence of any commercial or financial relationships that could be construed as a potential conflict of interest.

## Publisher's Note

All claims expressed in this article are solely those of the authors and do not necessarily represent those of their affiliated organizations, or those of the publisher, the editors and the reviewers. Any product that may be evaluated in this article, or claim that may be made by its manufacturer, is not guaranteed or endorsed by the publisher.
